# Capillary Bronchitis

**Published:** 1901-03

**Authors:** 


### Capillary Bronchitis.

℞ Vini antimon.,

Spir. chlorofomi....................aa ℥ iv.

Spir. ammon. arom.................... ℥ i.

Liq. ammon. acet.................... ℥ ij.

Aquæ..............................ad ℥ viij.

M. S. Tablespoonful every hour.

